# Exploring Perceptions and Experiences of Food Allergy among New Canadians from Asia

**DOI:** 10.1155/2014/964504

**Published:** 2014-06-04

**Authors:** Stephanie K. Lu, Susan J. Elliott, Ann E. Clarke

**Affiliations:** ^1^School of Public Health and Health Systems, University of Waterloo, 200 University Avenue West, Waterloo, ON, Canada N2L 3G1; ^2^Department of Medicine, University of Calgary, Calgary, AB, Canada T2N 1N4

## Abstract

*Introduction*. In Canada, perceived prevalence of food allergy surpasses systematic estimates. Canadian immigrants have been found more likely to rate the risk of food allergy as “high” compared to nonimmigrants. *Methods*. Qualitative interviews were conducted with 3 key informants and 18 allergic individuals of East and Southeast Asian descent in order to capture their lived experience with food allergies. *Results*. Participants found food allergies to be more common in Canada than in Asia. Participants also agreed that having a food allergy is more manageable in Canada as a result of the policy environment (e.g., food labelling and school policies). In addition, participants had dealt with skepticism and disbelief about their food allergy in Asia, resulting in social exclusion and impacting quality of life. *Discussion*. Findings demonstrate the need to recognize the varied impacts and experiences of food allergy among new Canadians, given that immigrants represent a large and growing proportion of the Canadian population.

## 1. Introduction

Food allergy policies in public places and stories in the popular press are evidence of the rising awareness of food allergies as an important public health risk [1]. In its most severe form, IgE-mediated food allergies can lead to anaphylaxis, which is potentially fatal. Internationally, food allergy prevalence varies widely with age and geography. For example, self-reported prevalence in the US is 9.1% (8% for children) and 5.3% for respondents with a physician diagnosis [2, 3]. In Canada, overall prevalence rates are around 7% [4]. The Canadian estimates, however, were based on a nonrepresentative sample that underrepresented vulnerable populations (i.e., low income families, immigrants, and Aboriginal Peoples) [5, 6]; a second national survey undertaken to address this limitation indicated that the prevalence of self-reported food allergy for immigrants who have lived in Canada for less than 10 years (herein referred to as “new Canadians”) was substantially lower than their Canadian born counterparts: 3.2% [7]. These researchers also investigated perceived prevalence of food allergy in the same populations and found that perceived prevalence was observed to surpass systematic estimates by up to 30% [8]. The gap between perceived and actual risk of food allergy suggests that the public's perception of true risk of allergy is, in fact, inflated [9]. Moreover, new Canadians were found more likely to rate the risk of food allergy as “high” compared to nonimmigrants or immigrants who have lived in Canada for over 10 years [9], despite the substantial differences in measured prevalence.

In light of the above, this paper used a qualitative research design to unpack the perceptions and experiences of new Canadians directly affected by food allergy by addressing the following objectives: (1) to understand how new Canadians perceive food allergies and their associated risks and (2) to investigate how new Canadians manage and cope with food allergies.

## 2. Methods

Waterloo Region, Ontario, Canada, was chosen as the research site as it is one of the fastest-growing areas in the province with the 9th highest proportion of visible minorities of all Census Metropolitan Areas across Canada [10].

Qualitative methods were chosen in order to privilege the voices of those affected. Qualitative methods have been used with several populations at risk of anaphylaxis: children [11–13], adolescents [14, 15], their parents [13, 14, 16], allergists [17], and the general public [18, 19].

In-depth interviews were conducted in two stages. First, key informants (an allergist, public health nutritionist, and public health planner) recruited through telephone and e-mail were interviewed in person (averaging 30 minutes) as representatives of key constituencies able to provide an information-rich connection to the research topic [20], as well as access to the wider food allergies community. Service providers and health workers have been shown to be rich and largely untapped sources of information with important insights to issues of health and migration [21]. Each interview covered topics specific to the key informant's observations and knowledge of food allergies from their professional work experience.

Second, in-depth interviews with affected individuals were conducted with new Canadians who either had a physician-diagnosed food allergy of their own or were a parent of a food allergic child. The focus was on individuals of East and Southeast Asian descent as 50% of the immigrant participants in the Canadian national prevalence survey were born in an Asian country [4, 5]. East and Southeast Asians are also two of the most common visible minorities in Waterloo Region [10]. Participants (*n* = 18) were recruited using Kijiji (an online network for posting local classified advertisements), the interviewed key informants, and contacts made with local cultural centres. Interviews averaged 30 minutes in length and were conducted (Nov. 2012 to Jan. 2013) until saturation was reached (i.e., until no new themes emerged from the interviews) [22]. Participants were also asked to complete a short demographic survey to gather additional information on their food allergy and length of time in Canada. 78% of the sample was female and 72% of all participants were under 30 years old (Table 1). Just over half the participants have lived in Canada for less than 10 years (*n* = 10). The most common self-reported allergen was shellfish (*n* = 12) and the majority of participants were over 5 years old when their allergy was diagnosed (*n* = 15).

Each interview was conducted by the primary author and audio-recorded for transcription and subsequent thematic analysis using QSR International's NVivo 9. A theme code set was created both deductively (based on the research objectives and interview guides) and inductively (themes emerging from the interview transcripts). Coding was assessed for inter- and intra-rater reliability [22], achieving 81% agreement with a second coder at the graduate level. Key themes that were identified were those that appeared with the greatest frequency as discrete units of text in the interview transcripts. Ethics clearance was received from the University of Waterloo.

## 3. Results

### 3.1. Key Informants

Three key themes emerged from the key informant interviews, the first being appropriate and informed diagnosis. New Canadians are concerned with receiving confirmation of their allergy and getting reassurance that their problem is not life-threatening. While the diagnosis process is straightforward, it can be difficult to educate new Canadians about how to avoid a reaction as well as the importance of using epinephrine in the form of an Epi-Pen:

Understanding what the issue is, is tough enough for patients that have grown up in Canada. To really appreciate and understand what a food allergy is, what it involves, and the potential risks, I think for immigrants it is even tougher because in most countries where they have immigrated from, allergy didn't exist. Or if it did exist, it was a really, really small issue. (Key Informant 1)

The second theme that emerged is the challenge of shaping a safe school environment for food allergic children. Placing bans on foods in schools can be very restrictive, making it difficult for people to find food alternatives, especially if cost is a concern. A restrictive diet can also make it more difficult to ensure that children are meeting nutritional needs:

It becomes very restrictive for the students who aren't allergic. So it is really concerning when foods are starting to be banned from the classroom, because soon, if there are more and more allergic students, many foods will be banned and it's very hard for people to find those foods and for there to be foods that are within the right price range, especially for people with lower income. So this is a major issue, and we really strive towards educating that it should be an allergy* safe* environment as opposed to an allergy* free* environment, because it really, in our opinion, is almost impossible to do that. (Key Informant 2)

Furthermore, studies have shown that food allergic children have been known to experience stigma and/or social exclusion, particularly in the school setting. How allergic children are being perceived by their peers, especially if an incident gets reported in the media, was raised by key informants:

I was really disturbed by the fact that you know [a story about an allergic child] was in the paper, and it was naming the school and the age of the child and the parent, so people know who this child is… I don't think it is fair to anyone involved, but it is definitely not fair to the child and we don't want to be causing that, so I just want to make it as normal as possible for them. (Key Informant 2)

### 3.2. Food Allergic New Canadians

Four key themes emerged from interviews with affected individuals: perceived prevalence, perceived etiology, management and coping, and quality of life. Initially, however, participants were asked to describe the emotions and feelings they experienced when they had their first allergic reaction. Many were surprised or even shocked to learn of their allergy (*n* = 12):

Yeah, I was actually quite surprised and so were my parents because we had been around people with allergies before, like my friends and stuff, but none of them had such a severe reaction… And especially because it was so unexpected at such a young age. I don't know, it is not common, so yeah, surprised was one thing. Scared was another. (Participant 15:* female raised in Singapore and Japan, <5 years in Canada, allergic to peanut and shellfish*)

Participants also felt disappointment (*n* = 7) and even sadness (*n* = 1) when they were diagnosed because they either found it difficult to give up a kind of food they liked or were upset that previous allergic reactions could have been prevented:

I would just feel sad, like I just think that it is a kind of sickness. I just feel it is a misfortune right, why do we have a son that has food allergy? Why other people don't have such problem, right? I just feel it is sad. I just kind of accept the fact. I have to face it, right? (Participant 4:* female from China, <10 years in Canada, son is allergic to peanuts and beans*)

One of the predominant perceptions regarding food allergies is that prevalence is lower in Asia than it is in North America [23]. Current evidence supports this perception, although it is speculated that rates of prevalence in more modernized regions of Asia, such as Hong Kong, are comparable to rates in North America [24, 25]. This perception was also held by participants, as 83% of them thought that food allergies were less common in their birth place:

Well I realized especially like in grade four and grade five, all my friends could not eat peanuts, so we couldn't even bring peanuts to school, so that is when I realized, oh food allergies are pretty common here in Canada. Whereas I never really thought about it back in the Philippines. (Participant 7:* male from Philippines, <20 years in Canada, allergic to shrimp and shellfish*)

It is a lot rarer in China. Over here there is a lot of emphasis [on it], like one of the first things you learn in school is don't bring any peanuts. You don't find that in China. (Participant 12:* female from China, <20 years in Canada, allergic to shrimp and prawns*)

78% of participants could not speculate on the cause of their allergy. However, when asked to speculate on the etiology of food allergy more generally, they cited examples related to diet, genetics, and the hygiene hypothesis. For example:

It might be because Chinese people eat everything, so from their ancestry they kind of don't have those allergies, because they start to eat anything like from years ago, but here people only eat certain kinds of food. They don't eat animals, like dogs and stuff, and so it is really hard to say. I don't know. (Participant 14:* female from China, <5 years in Canada, allergic to peanuts, beans, fish, and shrimp*)

Participants also found it harder to manage their allergy in their birth place (Table 2). Participants attributed this difference to lower levels of awareness and, subsequently, less respect and understanding of their needs for accommodation.

Food allergy impacts the quality of life of not only the allergic individual, but also their family. 39% of respondents reported that the impact of the food allergy on their quality of life was “negligible” (Table 3); that is, they adapted quickly to their food allergy and never felt any serious disappointment from being allergic. More participants (61%) felt that their allergy had a “noticeable” impact on their quality of life as it took some time to accept the allergy and learn how to manage everyday life. The allergy often went from being a nuisance to just a fact of life. Three participants reported being significantly impacted by the allergy; two had multiple allergies and one had tried various remedies, both Chinese and Western medicines, to alleviate symptoms. A third who reported significant quality of life impacts had been teased by family and friends:

My parents started helping me to treat my allergy back when I was fifteen or sixteen. I started eating this one particular thing called bird's nest. It is like a drink that is similar to ginseng. It is supposed to make your metabolism better. Every day I was drinking it. I wouldn't say for sure that I got better because of it. (Participant 1:* female from Hong Kong, <10 years in Canada, allergic to lychee and lobster*)

My mom, she is not allergic to anything, so she is always like “why are you allergic?” There is a phrase that she says [often]. She always says my sister will eat anything she cooks, but I am always the weird kid that [she] always has to be careful with, [because there are] so many limitations to the food that I eat. So it is just kind of annoying at times. (Participant 3:* male raised in Hong Kong, <20 years in Canada, allergic to shellfish and peanut*)

The presence of a strong support system can make a positive difference in coping. 72% of participants said their family and friends are supportive because they understand the severity of their allergy and are willing to make accommodations (Table 4). However, 39% of participants reported that their family was initially skeptical of their food allergy:

A lot of people are skeptical. My relatives [will say something] like “are you sure that you are allergic to it?” “Maybe you should try without knowing it and see if it is a psychological thing.” But I actually proved it to them many times that I am allergic. It is actually about my physical reaction. (Participant 3:* male raised in Hong Kong, <20 years in Canada, allergic to shellfish and peanut*)

Participants had to “prove” their allergy to their relatives by showing their physical reaction when they consumed an allergen.

Misinformed perceptions about oral immunotherapy were also raised, as participants had experienced situations in which they were encouraged by family members to eat an allergen. The thinking was that regular exposure to the allergen would eventually cure them of their condition:

[My dad] is like just keep eating, because whenever there were shrimps or like at home, my dad would always kind of force me to eat it, and he would be just like “oh it is all in your head. You can like overcome it if you just slowly like try to build immunity against it”… But I kind of didn't want to do it, just because I was afraid of getting a reaction. (Participant 7:* male from Philippines, <20 years in Canada, allergic to shrimp and shellfish*)

Interestingly, some participants had no prior knowledge of what a food allergy was, even if they had one, until they came to Canada:

R (R refers to the question asked by the researcher): When you were growing up were you familiar with what food allergies are? P (P refers to the participant's response): No, I actually wasn't. I didn't even know anyone who had allergies. It was only when I came to Canada that I had heard that people have allergies to peanuts and stuff like that. I didn't know how severe it can get, that people can actually get it, and so forth. (Participant 11:* male from Thailand, <20 years in Canada, allergic to fish and shellfish*)

Further, even if they were familiar, there was no mechanism in their native language to describe it. For example, 28% of participants could say “food allergy” in Chinese (Mandarin and Cantonese), but there was no equivalent Chinese term for “anaphylaxis.” The same observation was made by participants who spoke Malay, Thai, and Japanese. It was speculated by some participants that familiarity with food allergies in Asia may increase over time, but at this point it has not been recognized as a medical condition, at least not in the general population.

A key aspect of managing and coping with food allergies is information. Approximately 56% of participants said that their physician was their go-to source for information. Three of the four mothers with an allergic child identified their allergist as being extremely helpful. Community and public health services were mentioned less frequently (14% of total mentions).

Personal internet research was the preferred method for learning more about food allergies with 67% of participants having conducted internet searches at least once:

…thank god we have internet. A lot of information is on the internet. We have spent lots of time doing that… I can't remember the website I went to, but I do a lot of research. I try to dig into why he has this allergy. I have spent tons of time trying to figure that out. Unfortunately, I haven't found out why. (Participant 4:* female from China, <10 years in Canada, son is allergic to peanuts and beans*)

## 4. Discussion

Shellfish was the most common self-reported allergen amongst participants (Table 1). This finding is consistent with studies from Asia where shellfish allergy predominates. In contrast, the risk of peanut and tree nut allergy is much higher in individuals from Western countries [5, 26–28]. While the influence of birth place in development of shellfish allergy is still unknown, it is likely that multiple environmental factors are contributing to this phenomenon, including gender, living environment (urban versus rural), and age of intake [5]. Researchers are only beginning to find potential links between age of fish intake and allergic disease that may also be the case of shellfish allergy [28, 29]. These links are further complicated by genetic predisposition [30].

Interestingly, twelve participants were 10 years or older when diagnosed (Table 1). This is in sharp contrast to the high rates of allergic children in North America who are diagnosed at a much younger age (between 2 and 4 years) [3]. While reasons for this are unclear, contributing factors may include a lower level of awareness in new Canadians and limited availability of epinephrine autoinjectors in Asia [30, 31]. In countries such as the Philippines, Epi-Pens are not even available due to cost constraints [30]. Limited accessibility to health care in parts of Asia may partially explain why several participants never opted to have their food allergy confirmed by a doctor, a challenge to determining prevalence based on self-report.

These findings support the hypothesis that new Canadians may have a lower level of awareness of food allergies and, hence, their inflated perception of risk. The difference in their level of awareness was evident in discussions that touched on their first allergic experience, the etiology of food allergies, and the lack of policies in their birth place to protect them in public places. Furthermore, similar findings have been found in the reverse situation; parents of expatriate children with a food allergy in Singapore reported that differences in food preparation and food labeling in Asia caused the greatest rise in stress levels after moving to Singapore [32]. Most schools in Asia do not take precautionary measures to protect food allergic children and legislature on food labeling is rare [32]. By understanding factors that influence risk perception such as familiarity and personal ability to affect risk, policy makers can anticipate how the public will respond [33].

Furthermore, health professionals should be aware that there is a wide variation in the quality of information on food allergy distributed to patients when diagnosed overseas, if they receive any information at all. The internet was the preferred source of information reported. Hence, health professionals and leaders in food allergy advocacy should be encouraged to tailor comprehensive, online resources that are accessible to new Canadians.

In terms of management and coping, participants relied on food labels and prior experience with allergens to stay safe. Unsupervised oral immunotherapy, however, emerged as an unexpected theme. This is concerning as oral immunotherapy is not yet widely accepted as a treatment [34, 35]. Moreover, this practice put participants in an uncomfortable situation, which could have possibly led to accidental exposure and/or issues of management anxiety.

Social isolation/exclusion also emerged as participants who had lived with their food allergy in Asia expressed frustration as they fended for themselves, especially if they were the only one in their social circle with a food allergy.

Qualitative research studies are by definition constrained by sample size but allow for a deeper exploration of lived experience. The reliance on self-reported food allergy in this study is typical of research in this field but does present a potential bias on the part of respondents. Further, not all countries in East and Southeast Asia were represented in the sample, so transferability of research findings to other regions may not be possible. Given the level of diversity within Asia itself, we recognize that the treatment of our participants as a homogenous group is a limitation of this study.

## 5. Conclusions

These findings indicate that food allergy among new Canadians is an important (and potentially growing) public health issue deserving of attention, given the reported impacts on social isolation/exclusion and quality of life, as well as the dearth of resources available. This exploratory research raises several issues deserving of more research: health literacy levels in immigrant populations, the wider variety of allergens that immigrants may be reacting to, and further research to understand the impacts of food allergies in other ethnocultural groups in Canada as immigrants represent a large and growing proportion of the Canadian population. Subsequently, further research will be needed to inform and facilitate change at the policy level in order to protect allergic individuals in Canada and abroad.

## Figures and Tables

**Table 1:** Participant characteristics (*n* = 18).

Participant characteristics		Number of participants (%)
Gender	Male	4 (22)
	Female	14 (78)

Age group	18–21	4 (22)
	22–25	7 (39)
	26–30	2 (11)
	31–40	3 (17)
	41–50	2 (11)

Birth place	China	5 (28)
	Hong Kong	3 (17)
	Malaysia	1 (5)
	Philippines	4 (22)
	Thailand	2 (11)
	Vietnam	1 (5)
	Other^a^	2 (11)

Years lived in Canada	0–5	6 (33)
	6–10	4 (22)
	11–20	6 (33)
	21+	2 (11)

Allergic person in household	Self	14 (78)
	Child	4 (22)

Allergy^b^	Peanut	6 (33)
	Shellfish (including lobster, shrimp, and prawns)	12 (67)
	Fish	5 (27)
	Fruit (including lychee, durian, blueberries, and mango)	4 (22)
	Beans	2 (11)
	Egg	2 (11)
	Wheat	1 (5)
	Milk	1 (5)

Age of diagnosis (years)	0-1	2 (11)
	2–5	1 (5)
	6–10	3 (17)
	11–15	7 (39)
	16–20	5 (28)

^a^Participant was raised, but not born, in East or Southeast Asia.

^b^Sum is not equal to number of participants due to multiple responses.

**Table 2:** Obstacles to managing food allergy in birth place.

Obstacle	Number of participants (% of the total)	Mentions (% of the total)
Different eating habits	3 (17)	4 (18)
Different attitudes towards food	2 (11)	2 (9)
Less accommodative (in restaurants, schools, etc.)	7 (39)	9 (41)
Different health safety standards	5 (28)	6 (27)
Cost	1 (5)	1 (5)

Total	18 (100)	22 (100)

**Table 3:** Impact of food allergy on quality of life in Canada.

Impact level	Number of participants (% of the total)	Mentions (% of the total)
Negligible	7 (39)	18 (46)
Noticeable	11 (61)	14 (36)
Significant	3 (17)	7 (18)

Total	18^a^ (100)	39 (100)

^a^This is not equal to the sum of the numbers in the column due to multiple responses.

**Table 4:** Family and friends' attitudes towards food allergy.

Attitude	Family		Friends	
	Number of participants (% of the total)	Mentions (% of the total)	Number of participants (% of the total)	Mentions (% of the total)
Surprise	4 (22)	7 (14)	1 (22)	1 (3)
Skeptical	7 (39)	8 (16)	3 (17)	4 (13)
Scared	2 (11)	2 (4)	0	0
Quick acceptance	4 (22)	4 (8)	3 (17)	3 (10)
Slow acceptance	5 (28)	8 (16)	2 (11)	2 (6)
Disappointment	0	0	1 (6)	1 (3)
Supportive/cautious	13 (72)	19 (39)	13 (72)	20 (65)
Other	1 (6)	1 (2)	0	0

Total	18^a^ (100)	49 (100)	18^a^ (100)	31 (100)

^a^This is not equal to the sum of the numbers in the column due to multiple responses.
